# Supplementary material for: The COVID-19 Mortality Rate Is Associated with Illiteracy, Age, and Air Pollution in Urban Neighborhoods: A Spatiotemporal Cross-Sectional Analysis
Source: Trop Med Infect Dis. 2023 Jan 26;8(2):85. doi: 10.3390/tropicalmed8020085 (PMC9962969; doi:10.3390/tropicalmed8020085)
Supplement: Supplementary file 1 [file tropicalmed-08-00085-s001.zip › Supplementary ‎File S8.pdf]

Summary of OLS Results - Model Variables

Variable	Coefficient [a]	StdError	t-Statistic	Probability [b]	Robust_SE	Robust_t	Robust_Pr [b]	VIF [c]
Intercept	-41.632449	20.151766	-2.065945	0.039573*	22.029342	-1.889864	0.059616	--------
V11	0.984721	0.247426	3.979857	0.000092*	0.266428	3.696018	0.000266*	1.067274
V13	0.040497	0.007752	5.224144	0.000001*	0.007683	5.271278	0.000000*	2.580485
V16	0.173753	0.029002	5.991168	0.000000*	0.026012	6.679628	0.000000*	1.592339
V24	1.859288	0.508727	3.654784	0.000309*	0.586948	3.167721	0.001686*	1.202649
V26	0.167494	0.029794	5.621789	0.000000*	0.031512	5.315314	0.000000*	2.311148

OLS Diagnostics

Input Features:	NormalizedData_For_OLS_	Dependent Variable:	TOTALEBS
Number of Observations:	350	Akaike's Information Criterion (AICc) [d]:	3455.815461
Multiple R-Squared [d]:	0.568256	Adjusted R-Squared [d]:	0.561981
Joint F-Statistic [e]:	90.553781	Prob(>F), (5,344) degrees of freedom:	0.000000*
Joint Wald Statistic [e]:	369.500520	Prob(>chi-squared), (5) degrees of freedom:	0.000000*
Koenker (BP) Statistic [f]:	11.641370	Prob(>chi-squared), (5) degrees of freedom:	0.040046*
Jarque-Bera Statistic [g]:	558.961143	Prob(>chi-squared), (2) degrees of freedom:	0.000000*

Notes on Interpretation

* An asterisk next to a number indicates a statistically significant p-value (p < 0.01).

[a] Coefficient: Represents the strength and type of relationship between each explanatory variable and the dependent variable.

[b] Probability and Robust Probability (Robust_Pr): Asterisk (*) indicates a coefficient is statistically significant (p < 0.01); if the Koenker (BP) Statistic [f] is statistically significant, use the Robust Probability column (Robust_Pr) to determine coefficient significance.

[c] Variance Inflation Factor (VIF): Large Variance Inflation Factor (VIF) values (> 7.5) indicate redundancy among explanatory variables.

[d] R-Squared and Akaike's Information Criterion (AICc): Measures of model fit/performance.

[e] Joint F and Wald Statistics: Asterisk (*) indicates overall model significance (p < 0.01); if the Koenker (BP) Statistic [f] is statistically significant, use the Wald Statistic to determine overall model significance.

[f] Koenker (BP) Statistic: When this test is statistically significant (p < 0.01), the relationships modeled are not consistent (either due to non-stationarity or heteroskedasticity). You should rely on the Robust Probabilities (Robust_Pr) to determine coefficient significance and on the Wald Statistic to determine overall model significance.

[g] Jarque-Bera Statistic: When this test is statistically significant (p < 0.01) model predictions are biased (the residuals are not normally distributed).

Variable Distributions and Relationships

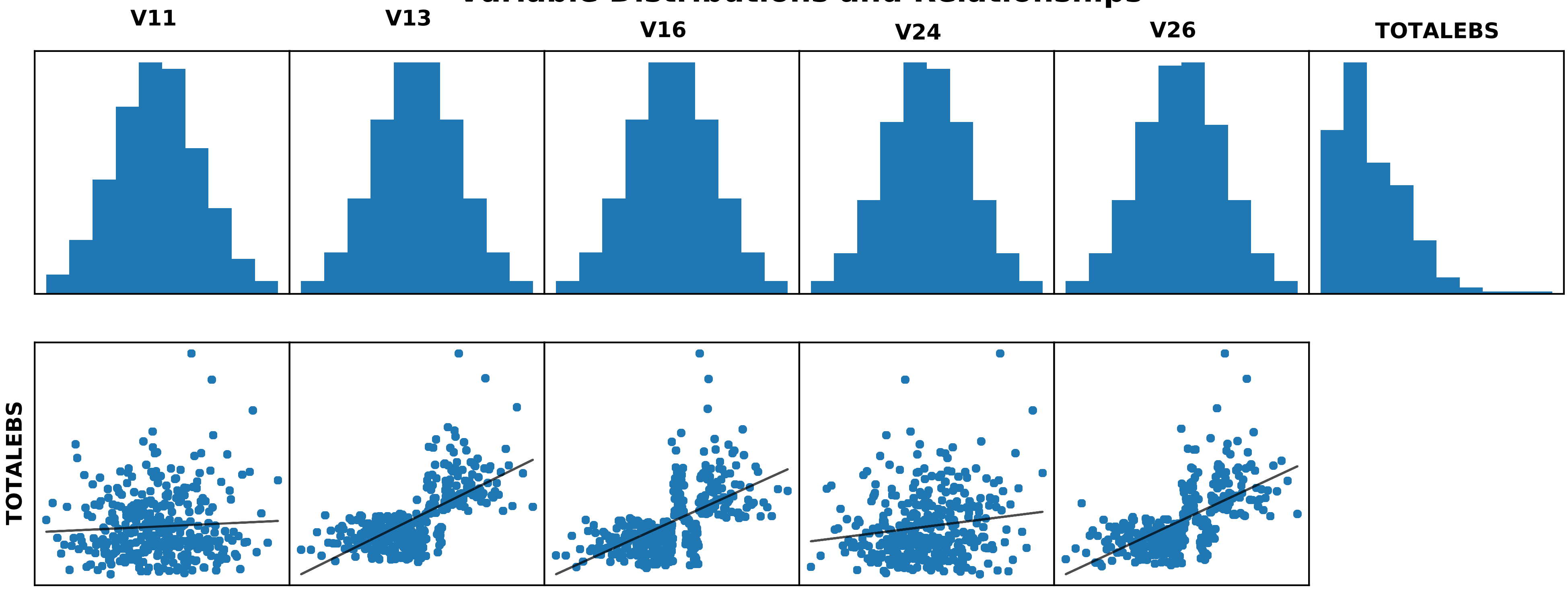

The above graphs are Histograms and Scatterplots for each explanatory variable and the dependent variable. The histograms show how each variable is distributed. OLS does not require variables to be normally distributed. However, if you are having trouble finding a properly-specified model, you can try transforming strongly skewed variables to see if you get a better result.

Each scatterplot depicts the relationship between an explanatory variable and the dependent variable. Strong relationships appear as diagonals and the direction of the slant indicates if the relationship is positive or negative. Try transforming your variables if you detect any non-linear relationships. For more information see the Regression Analysis Basics documentation.

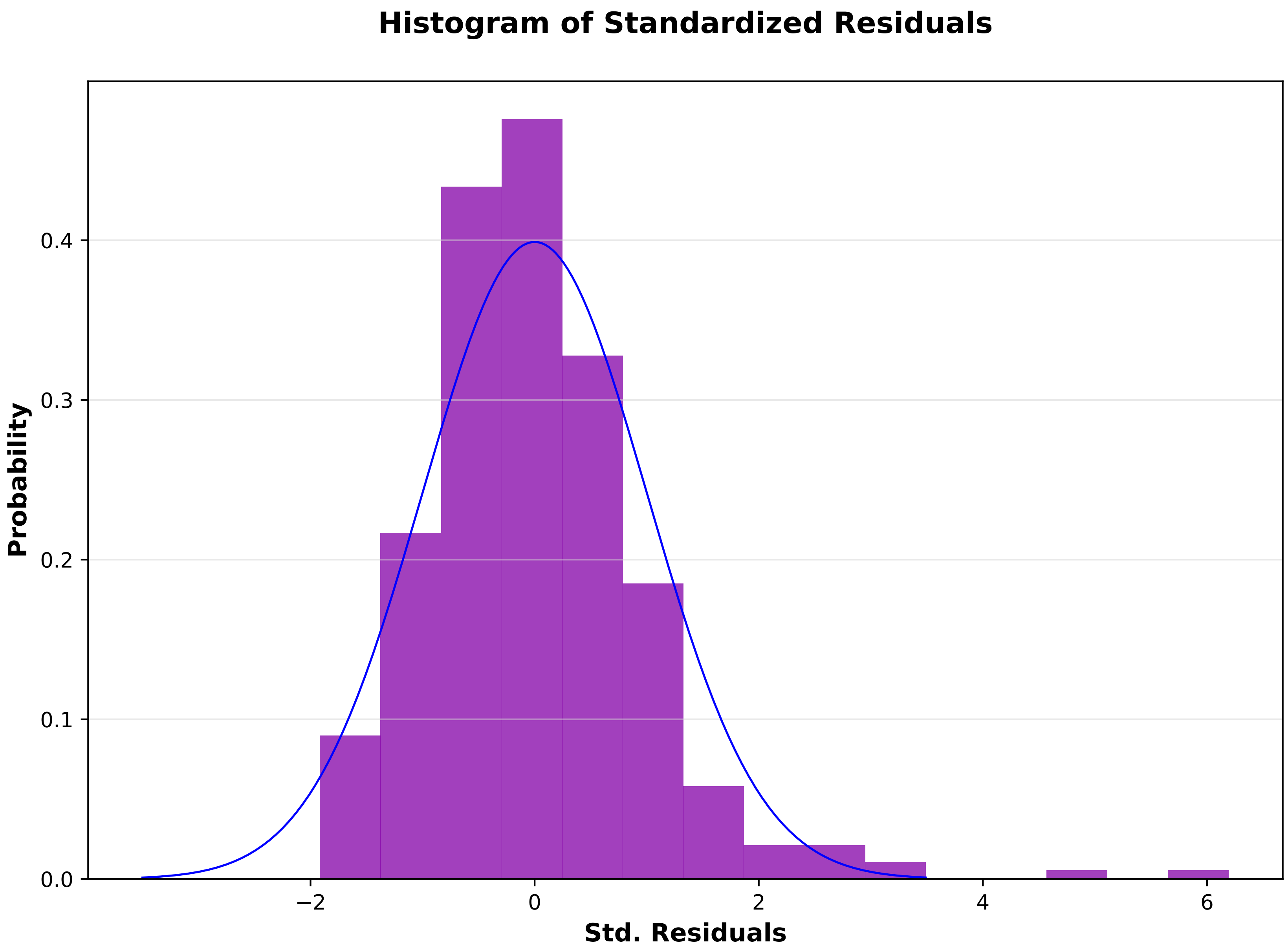

Ideally the histogram of your residuals would match the normal curve, indicated above in blue. If the histogram looks very different from the normal curve, you may have a biased model. If this bias is significant it will also be represented by a statistically significant Jarque-Bera p-value (*).

Residual vs. Predicted Plot

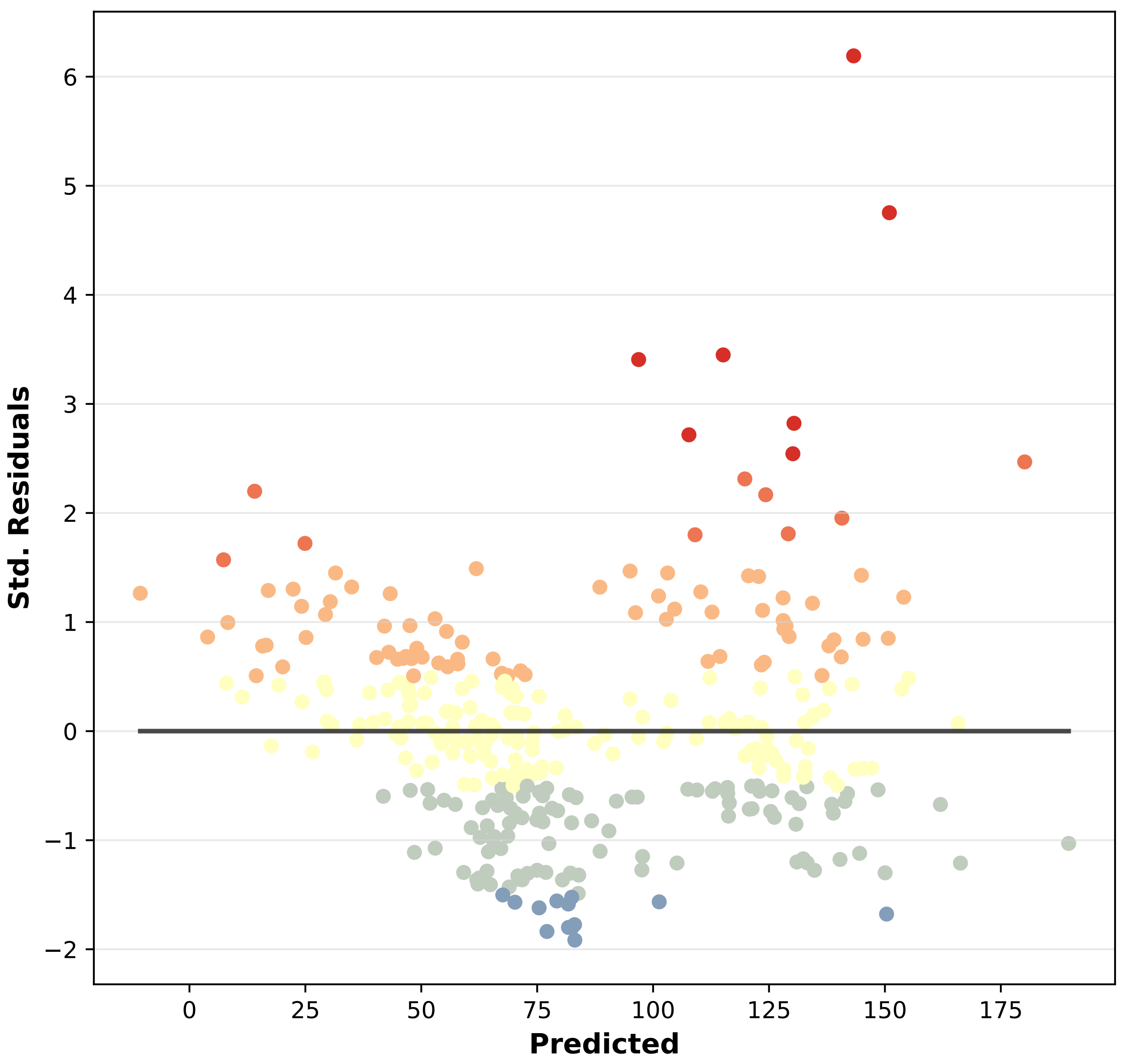

This is a graph of residuals (model over and under predictions) in relation to predicted dependent variable values. For a properly specified model, this scatterplot will have little structure, and look random (see graph on the right). If there is a structure to this plot, the type of structure may be a valuable clue to help you figure out what's going on.

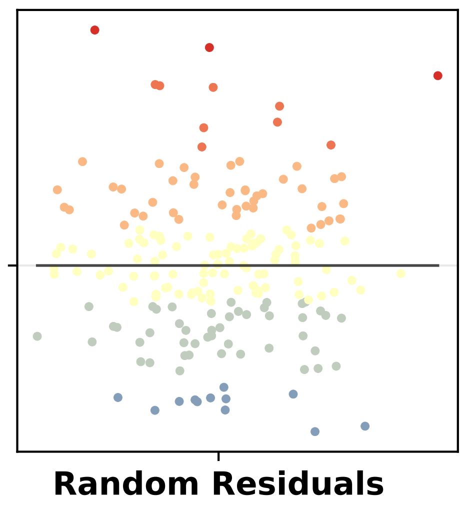

Ordinary Least Squares Parameters

Parameter Name	Input Value
Input Features	NormalizedData_For_OLS_GWR_MGWR
Unique ID Field	NeighID
Output Feature Class	
Dependent Variable	TOTALEBS
Explanatory Variables	V11<br>V13<br>V16<br>V24<br>V26
Selection Set	False
